# Traumatic Brain Injury Induces Senescence in Brain Microvasculature

**DOI:** 10.3390/biom16030359

**Published:** 2026-02-28

**Authors:** Tejal Shreeya, Zsófia R. Hernádi, Zsolt K. Bali, Nóra Bruszt, István Hernádi, Bálint Fazekas, Krisztina Amrein, Endre Czeiter, Csilla Fazakas, Imola Wilhelm, István A. Krizbai, Attila E. Farkas

**Affiliations:** 1 Institute of Biophysics, HUN-REN Biological Research Centre, H-6726 Szeged, Hungary; shreeya.tejal@brc.hu (T.S.);; 2 Doctoral School of Experimental and Preventive Medicine, University of Szeged, H-6720 Szeged, Hungary; 3 Grastyán Endre Translational Research Centre, University of Pécs, H-7624 Pécs, Hungary; 4 Translational Neuroscience Research Group, Centre for Neuroscience, Szentágothai Research Centre, University of Pécs, H-7624 Pécs, Hungary; 5 Institute of Physiology, Medical School, University of Pécs, H-7624 Pécs, Hungary; 6 Department of Neurobiology, University of Pécs, H-7624 Pécs, Hungary; 7 Department of Neurosurgery, Medical School, University of Pécs, H-7623 Pécs, Hungary; 8 Molecular Medicine Research Group, Szentágothai Research Centre, University of Pécs, H-7624 Pécs, Hungary; 9 HUN-REN–PTE Clinical Neuroscience MR Research Group, University of Pécs, H-7623 Pécs, Hungary; 10 Institute of Life Sciences, “Vasile Goldis” Western University of Arad, RO-310414 Arad, Romania; 11 Department of Cell Biology and Molecular Medicine, University of Szeged, H-6720 Szeged, Hungary

**Keywords:** traumatic brain injury, TBI, cellular senescence, γH2AX, neurovascular unit, NVU

## Abstract

Background: Traumatic brain injury (TBI) frequently leads to long-term neurological deficits. Recent research also implicates cellular senescence—a state of permanent cell cycle arrest driven by DNA damage—as a key contributor to neuroinflammation and cognitive decline. This study investigates the cell-type specificity of senescence within glial and vascular cells of the neurovascular unit (NVU) following experimental TBI in a rat model. Methods: Rats underwent various TBI scenarios, including single severe TBI (sTBI), single mild TBI (mTBI), repetitive mild TBI (rmTBI) and repetitive sham-operated control (rSham). Twenty-four hours or four weeks later, brains were harvested and brain sections were co-stained for γH2AX and cell type-specific markers. Immunofluorescence microscopy was used to comprehensively assess senescence in both glial and vascular cells of the NVU, specifically astrocytes, microglia, endothelial cells, and pericytes. Results: We observed acute increased astrocyte senescence in sTBI samples and microglial senescence in mTBI and sTBI samples in the neocortex, while endothelial cell senescence was significantly elevated in the neocortex of the sTBI group after four weeks. Pericytes did not exhibit significant signs of senescence at either time point. Conclusion: These findings demonstrate differential γH2AX labelling of NVU components following TBI, suggesting that vulnerability to TBI-induced senescence can be specific both to the cell type and the time after the injury. This has implications on therapies targeting senescent cells for mitigating the long-term consequences of TBI.

## 1. Introduction

Diffuse traumatic brain injury (TBI) is a leading cause of disability. Though TBI is more prevalent in athletes, victims of domestic abuse, and military personnel, it has become a frequently occurring condition in the general population, primarily attributable to motor vehicle collisions, occupational hazards, and accidental falls [1]. TBI is defined as a blow or jolt to the head—including brain concussions and subconcussive brain injuries—that disrupts neurological function, and may vary in severity and clinical presentation. This impact causes tissue deformation, leading to primary tissue damage affecting both the neural parenchyma and its vasculature. Primary tissue damage is often followed by secondary damage, including haemorrhage, brain swelling, neuroinflammation, neurodegeneration and blood–brain barrier (BBB) defects, some of which are potentially reversible [2]. TBI commonly leads to a wide array of neurological symptoms such as headaches, chronic fatigue, balance problems, nausea, cognitive and emotional symptoms, sleep abnormalities, neurodegeneration and dementia, some of which can be long lasting and irreversible [3]. Cellular senescence may play a key role in secondary damage after TBI. Mechanical and oxidative stress, neuroinflammation, and genotoxic damage can contribute to senescence, while accumulation of senescent cells can induce further inflammation contributing to inflammageing and consequently to premature progressive neurodegeneration and pathological cognitive ageing [4,5].

Cellular senescence is a multifaceted state of permanent and irreversible cell cycle arrest, first described in cell culture by Hayflick and Moorehead in 1961, and is also the first indication that cellular senescence contributes to ageing [6]. Senescence is characterised by morphological, cellular, and molecular changes, oxidative damage, resistance to apoptosis and hence sustained viability. In vivo senescence plays important roles in embryogenesis, adult tissue remodelling and wound healing, while its role in diseases can be detrimental or beneficial depending on the disease [7]. Senescence encompasses multiple molecular changes, many of which are also useful for detecting senescence. Among these, the earliest described alteration was the upregulation of senescence-associated β-galactosidase [8]. Changes in cell cycle regulatory mechanisms confer apoptosis resistance to senescent cells, and thus the upregulated levels of p21, p16^INK4a^ and p53 are commonly used as senescence markers [9]. Similar significant changes can be observed in anti-apoptotic pathways and in the secretory function of senescent cells, resulting in a characteristic senescence-associated secretory phenotype (SASP) [10,11]. Important drivers of ageing and cellular senescence appear to be double-stranded DNA (dsDNA) breakage and the loss of DNA repair capability [12], which might both play central roles in TBI-induced premature brain ageing and consequent progressive neurodegeneration. DNA damage in cells triggers repair mechanisms, including the phosphorylation of the core histone protein H2AX at the serine 139 position. This phosphorylated protein is called γH2AX, and is also considered a robust marker of double-stranded DNA breaks and cellular senescence [9,13].

Cellular senescence is an essential element of brain ageing which affects all aspects of life; in addition to cognition, it affects sensory as well as motor functions. The senescence of neurons—surprising as neurons are postmitotic cells—is considered to contribute to brain ageing [14]. TBI is linked to accelerated ageing and senescence in both experimental animals and humans [5,15]. In response to TBI, numerous pathways related to inflammatory and senescence signalisation are activated in the juvenile mouse brain [16]. In the ageing brain, TBI can exacerbate brain ageing and neurodegenerative diseases [4].

Neural function is supported by multiple cell types in the brain. Learning how each of these cell types contributes to TBI-induced senescence could give useful insight for future therapies. The cells of the neurovascular unit (NVU) ensure nutrient supply and oxygen delivery to neural tissue, restrict harmful substances entering the brain, and remove waste products through neurovascular coupling and the BBB. In addition to neurons, the main cellular components of the NVU are endothelial cells, pericytes, microglia and astrocytes [17]. Endothelial cells line the vasculature and form the physical basis of the BBB, while pericytes play roles in the development and maintenance of the BBB as well as participating in blood flow regulation. The senescence of these vascular cells is accompanied by loss of BBB integrity. In vitro results suggest that senescence of endothelial cells and pericytes leads to BBB dysfunction [18]. In the ageing brain, BBB leakage can be observed in the hippocampus of healthy individuals around the age of fifty [19]. Microglia are the resident macrophages of the brain and migrate to the site of tissue injuries, and thus are exposed to the microenvironment of the injured neural tissue, which through oxidative and inflammatory processes can induce cellular senescence. Senescence leads to decreased microglia function, which plays an important role in accelerated brain ageing in neurodegenerative diseases [20]. Astrocytes are key supporters of neuronal function and also migrate to sites of injury in the brain and are involved in brain tissue regeneration [21]. Senescent astrocytes have been shown to have decreased neuroprotective capacity both in vitro and in vivo [22,23].

In the present study, we used immunofluorescence to label γH2AX co-stained with cell-type-specific markers of NVU components to investigate the cell type specificity of cellular senescence in brain sections derived from rats that previously underwent experimental TBI using the Marmarou weight drop method [24].

## 2. Materials and Methods

### 2.1. Animals

Altogether, 24 male Long Evans rats were used in the present study and were 5 months old at the time of the TBI intervention. The animals were housed in pairs in the minimal disease animal facility of the Szentágothai Research Centre, University of Pécs, Hungary. Temperature and humidity of the animal house was daily monitored. In the animal house, the lights were ON from 7 a.m. to 7 p.m., and the procedures were conducted during the light period. All procedures complied with the domestic and EU regulations (Decree No. 40/2013. (II. 14.) of the Hungarian Government and EU Directive 2010/63/EU). The experiments were approved by the Animal Welfare Committee of the University of Pécs and the National Scientific Ethical Committee on Animal Experimentation (ÁTET) at the Ministry of Agriculture (licence no. BA02/2000-69/2017).

### 2.2. Marmarou Weight Drop TBI Model

Rats were divided in 4 groups according to the severity of the injury, as follows: single severe TBI (sTBI, n = 6), single mild TBI (mTBI, n = 6), repetitive mild TBI (rmTBI, n = 6), repetitive sham-operated (control) rSham (n = 6). Each of these were further divided into two subgroups according to the survival time (24 h or 4 weeks), making 8 final groups of animals with equal sample size (n = 3) in each subgroup.

For the TBI procedure, the animals underwent an impact acceleration TBI method initially described by Foda and Marmarou [24] and which has also been applied in our laboratory [25,26,27].

First, animals were anaesthetised with isoflurane gas. Anaesthesia was induced for 5 min with 4% isoflurane (Forane, Abbott, Hungary) in 70% N_2_ and 30% O_2_ in an induction box, and rats were maintained under anaesthesia throughout the injury and surgical procedure. Rats were ventilated with 2–3% isoflurane in 70% N_2_ and 30% O_2_ (Inspira ASV, Harvard Apparatus, Holliston, MA, USA). Through the entire surgical procedure, physiologic parameters (oxygen saturation (SpO2), heart rate) of the animals were monitored by a pulse oximeter (MouseOx Plus, Starr Life Sciences Corp., Oakmont, PA, USA), while body temperature was monitored and kept at 37 °C by a homeothermic monitoring system (Harvard Apparatus, Holliston, MA, USA). All the monitored physiological parameters were within the normal ranges throughout all operations.

Once the anaesthesia was stabilised, animals were placed on a raised platform with a foam bed, their skull was exposed with a midline incision made in the skin, and a metallic disc was placed at the centre of the skull, between the lambda and bregma craniometric points. A 2 m long hollow plexiglass tube was placed just above the metallic disc, from which a 450 g weight was dropped onto the skull surface. The severity of the injury was dependent on the height from which the weight was dropped. In the case of sTBI, the weight was dropped from a height of 150 cm, while in the case of mTBI, the weight was dropped from a height of 25 cm. In the case of rmTBI, the mTBI procedure was repeated 5 times (once daily) on five consecutive days to mimic repetitive injuries that commonly occur during sports. In the sham-operated control (rSham) condition, all repeated surgical procedures except the weight drop were completed.

### 2.3. Neurological Screening

The four subgroups of animals with four weeks post-TBI survival were subjected to the modified neurological severity scoring (mNSS) test to assess possible residual neurological alterations. The mNSS is a composite motor and sensory test battery including the following tests: fore- and hindlimb flexion test, head deviation test, gait test, placement tests, proprioceptive test, beam balance test, and assessment of spinal and cranial nerve reflexes (pinna, cornea, startle) and abnormal movements (caused by, e.g., seizure, myoclonus, or dystonia) [28]. No training sessions were run before the assessment of the mNSS. The maximum composite mNSS score is 18, which represents severe abnormalities in all assessments. The score can be evaluated as mild deficit (1–6 points), moderate deficit (7–12 points) or severe deficit (13–18 points). For detailed scoring, see Supplementary Table S1. The mNSS scores of rats in different groups were compared using one-way Kruskal–Wallis test in jamovi [29,30].

### 2.4. Immunofluorescent Staining

To investigate the acute and chronic cellular effects of TBI at 24 h and four-week post injury time points, corresponding animals were euthanized with an overdose of pentobarbital anaesthetic (>400 mg/kg) and were transcardially perfused with saline and 4% paraformaldehyde (PFA) solution. Brains were postfixed overnight in 4% PFA. Then, 30 μm thick coronal sections from the midbrain region were sectioned using a vibratome (VT1000 S Vibrating blade microtome, Leica, Wetzlar, Germany) and stored in PBS until used for immunofluorescence staining. Antigen heat retrieval was performed in 20 mM Tris buffer, pH 8, at 85 °C for 20 min. Sections were permeabilised with 0.5% Triton X-100 (Sigma-Aldrich, St. Louis, MO, USA) in PBS (phosphate-buffered saline) for 20 min and blocked with 3% BSA (bovine serum albumin, VWR Life Science, Radnor, PA, USA) in PBS for 30 min. Primary antibodies (Table 1) were diluted in a blocking solution and the sections were incubated overnight at 4 °C on an orbital shaker. Cell type-specific antibodies were used to determine the senescence of neurovascular components: PECAM-1/CD31 for endothelial cells, Iba1 for microglia, GFAP for astrocytes, and PDGFRβ for pericytes. During this project, the rabbit anti-PECAM-1 antibody that was used for the 24 h samples was switched to a goat anti-PECAM-1 antibody that was used for the 4-week samples. Both PECAM-1 antibodies labelled the cell membrane of all microvascular endothelial cells and were enriched at tight junctions. Gamma H2AX antibody was used as a senescence marker for all cell types. Sections were then washed in PBS three times. Alexa-fluor-labelled secondary antibodies (Table 2) were used at a dilution of 1:500 in blocking solution for an hour at room temperature in the dark. Sections were washed, counterstained with a nuclear dye (Hoechst 33342; Sigma-Aldrich) for 10 min, washed with PBS, rinsed in water and mounted on a slide in FluoroMount-G (Invitrogen, Waltham, MA, USA).

### 2.5. Epifluorescence and Laser Confocal Microscopy

Samples were analysed using a fluorescent microscope (Axiovert Z1, Zeiss, Budapest, Hungary) equipped with super-resolution-capable laser scanning confocal microscopy (Stedycon, Abberior Instruments, Göttingen, Germany). Objectives used were 20× and 100×. The 20× epifluorescence images were acquired for easily identifiable glial cells like astrocytes and microglia, while 100× confocal images were necessary to reliably identify mural cells: endothelial cells and pericytes. Gamma H2AX-positive and total cell numbers were counted using ImageJ (version 1.54p) [31]. All well-defined cells in one 20× field of view were analysed for each animal, yielding an average of 151 and 421 cells in the case of astrocytes and microglia, respectively. Mural cells were counted in multiple 100× z-stacks until about 50 cells were found, and thus an average of 52 endothelial cells and 34 pericytes were analysed for each animal. Statistics were performed in Prism GraphPad 8.

## 3. Results

The study aimed to investigate cellular senescence within the NVU in response to TBI in a rat model.

**Figure 1:**
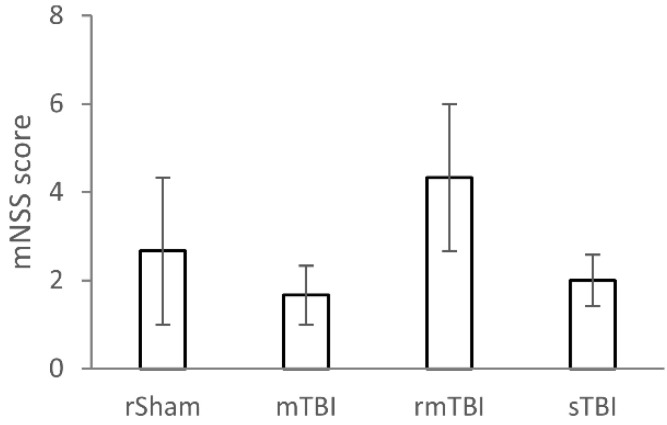
Modified neurological severity scoring (mNSS) of rats at 4 weeks after traumatic brain injury (TBI). The mNSS score is a composite of various motor and sensory tests, including fore- and hindlimb flexion test, beam balance test, gait test, and head deviation test. The mNSS scores were compared using one-way Kruskal–Wallis test, n = 3 rats/group. No significant differences were observed between the different TBI severity groups. Abbreviations of treatment groups: rSham: repetitive sham-operated (control); mTBI: mild TBI; rmTBI: repetitive mild TBI; sTBI: severe TBI.

TBI was induced using the Marmarou weight drop model, which was followed by the mNSS screening at the four-week time point after TBI (Figure 1). The tests showed no significant difference between the groups of animals in the mNSS test (χ^2^ = 1.625, df = 3, *p* = 0.654). All animals performed well in the neurological tests, as there were no deficits found in sensory tests, placements tests, gait tests, and reflex tests. In general, rats showed some mild levels of deficit only in the beam balance test; thus, the overall mNSS score represented the scores given in the beam balance test. Thus, according to the overall mNSS score, all animals exhibited some mild neurological deficits, including those in the rSham (control) injury group, which may be attributable to repeated anaesthesia.

Brains were harvested postmortem and were fixed and sectioned to 30 µm using a vibratome (see Section 2). Such thick sections were used because the short blood vessels in thinner sections are often not sufficient for reliable identification of endothelial cells and pericytes. Brain sections were co-stained with cell-type-specific markers and γH2AX, to detect double-stranded DNA (dsDNA) breaks that are characteristic to senescent cells. The parietal cortex lying directly under the impacted skull and the deeper region of the hippocampus were selected for analysis.

**Figure 2:**
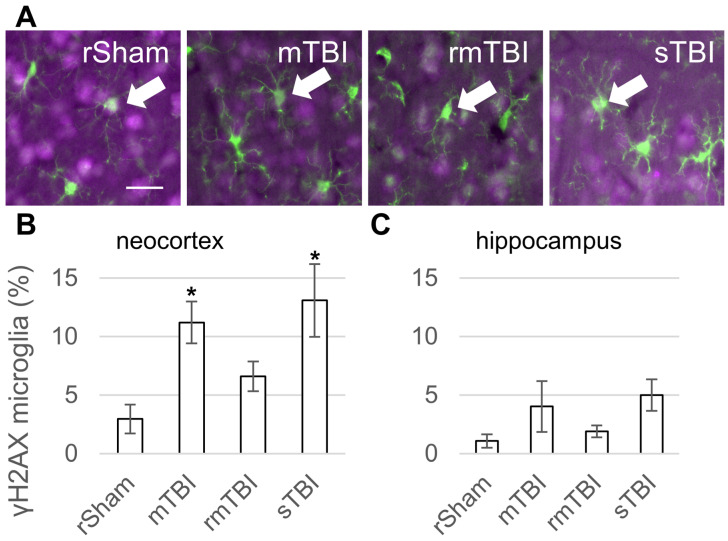
Microglia senescence 24 h after traumatic brain injury. (**A**) Senescence was detected in Iba1-positive (green) microglia through γH2AX (magenta) co-staining. The arrows mark γH2AX-positive senescent microglia in the neocortex. Scalebar is 25 µm. (**B**,**C**) The ratios of γH2AX-positive microglia were determined in the parietal neocortex and hippocampus 24 h post injury. Data is presented as a percentage of the total microglia number counted. ANOVA with Dunnett’s test, * *p* < 0.05, n = 3 rats/group. For abbreviations on treatment groups, see Figure 1.

Senescence was detected in Iba1-positive microglia through γH2AX co-staining; representative images are shown from the neocortex, where significant changes were observed (Figure 2A). The neocortex of TBI animals at 24 h showed a statistically significant increase in senescent microglia in the sTBI and mTBI groups, but not in rmTBI (Figure 2B). The hippocampus of TBI animals of the 24 h group showed an increase in the number of senescent cells, but this was not statistically significant (Figure 2C).

At four weeks post injury, we did not observe any γH2AX-positive astrocytes or microglia.

**Figure 3:**
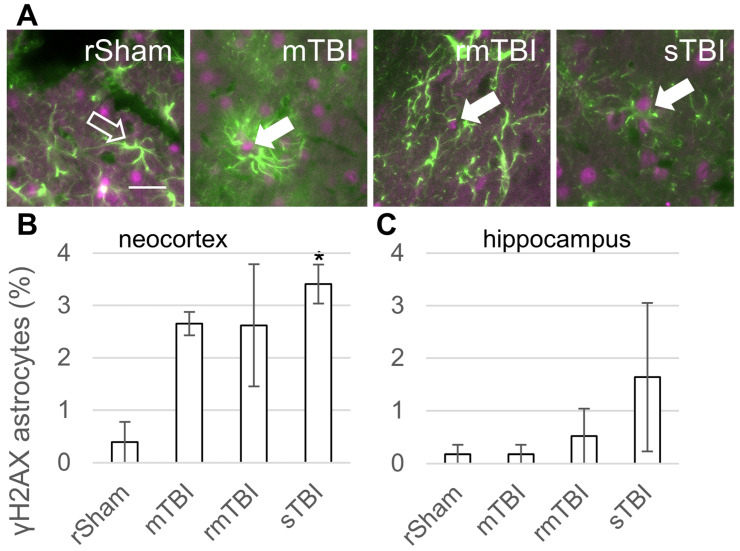
Astrocyte senescence 24 h after traumatic brain injury. (**A**) Senescent astrocytes were identified by γH2AX (magenta)/GFAP (green) co-staining. The arrows mark γH2AX-positive senescent astrocytes in representative images from the neocortex; arrow outline marks a negative astrocyte. Scalebar is 25 µm. (**B**,**C**) The ratios of γH2AX-positive astrocytes were determined in the parietal neocortex and hippocampus 24 h post injury. Data is presented as a percentage of the total astrocyte number counted. ANOVA with Dunnett’s test, * *p* < 0.05, n = 3 rats/group. For abbreviations on treatment groups, see Figure 1.

We observed γH2AX-positive senescent astrocytes 24 h after TBI (Figure 3A). The ratio of senescent astrocytes was smaller than in the case of microglia. At 24 h post injury, all TBI groups exhibited an increase in senescent astrocytes in the neocortex compared to the rSham group (Figure 3B). The prevalence of senescent astrocytes was highest in the sTBI group, followed by mTBI and rmTBI groups, although only the sTBI group was statistically significant. In the hippocampus, the sTBI group also showed senescent astrocytes, but this increase was not statistically significant (Figure 3C).

**Figure 4:**
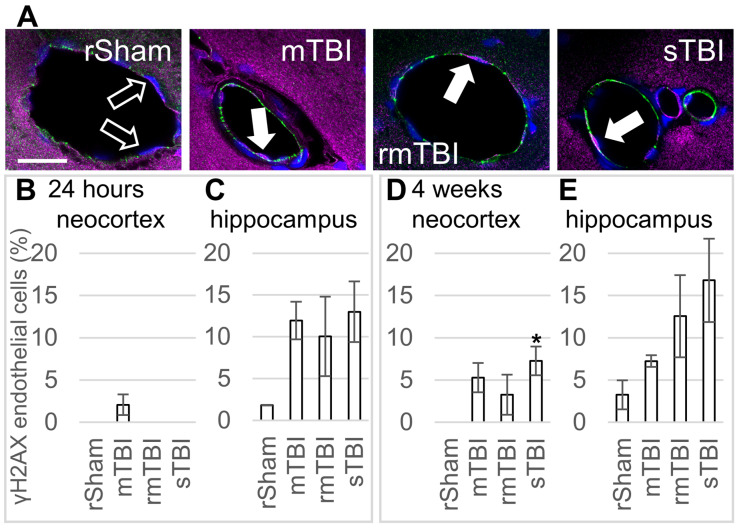
Endothelial senescence 24 h and 4 weeks after traumatic brain injury. (**A**) Senescence of PECAM-1-labelled (green) endothelial cells was detected by γH2AX (magenta) co-staining. Hoechst 33342-stained nuclei were blue. The arrows mark γH2AX-positive senescent endothelial cells in representative images from the neocortex 4 weeks after injury; arrow outline marks negative cells. Scalebar is 25 µm. (**B**,**C**) The ratios of γH2AX-positive endothelial cells were determined in the parietal neocortex and hippocampus 24 h post injury. (**D**,**E**) The ratios of γH2AX-positive endothelial cells in the parietal neocortex and hippocampus 4 weeks post injury. Data is presented as a percentage of the total endothelial cell number counted. ANOVA with Dunnett’s test, * *p* < 0.05, n = 3 rats/group.

Similar to the glial cells, senescence was identified in CD31/PECAM-1-positive endothelial cells through γH2AX co-staining (Figure 4A). However, in contrast to both astrocytes and microglia, 24 h after the injury, endothelial cells in the neocortex were comparatively less susceptible to senescence, with only the mTBI group showing a presence of senescent endothelial cells (Figure 4B). In contrast, the hippocampus showed a higher number of senescent endothelial cells in all TBI groups, though these changes were not statistically significant (Figure 4C). In the hippocampus, the sTBI group exhibited the highest number of senescent endothelial cells, followed by mTBI and rmTBI, in comparison to the rSham group.

On the other hand, 4 weeks after the injury, in the neocortex, all TBI groups showed increased senescent endothelial cell numbers, but only the increase in the sTBI group was statistically significant (Figure 4D). In the hippocampus, 4 weeks after the injury, all TBI groups had increased endothelial cell senescence, but these changes were not statistically significant (Figure 4E).

**Figure 5:**
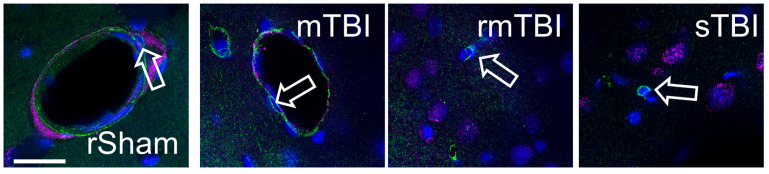
Pericytes did not become γH2AX-positive after TBI. We did not observe any senescent pericytes either 24 h or 4 weeks post-TBI using PDGFRβ (green) with γH2AX (magenta) co-staining. Arrow outlines mark negative pericytes 24 h after injury. Hoechst 33342-stained nuclei were blue. Arrow outlines point to pericytes; some unlabelled cells nearby are positive for γH2AX.

To detect senescent pericytes, the specific marker PDGFRβ was co-stained with senescence marker γH2AX. However, we found no senescent pericytes at 24 h or 4 weeks post injury (Figure 5).

## 4. Discussion

Recent research underlines the importance of inflammation and the resulting senescence in neurodegenerative processes and cognitive impairment following TBI [1,5]. Neurons are the most well studied cell type in TBI and other neurological pathologies. Much less is known about the cell types supporting neuron function—especially the cells of the brain microvasculature—under these conditions. As the NVU plays crucial roles in maintaining neuronal function in health and disease [32], we asked whether constituents of the NVU other than neurons show acute or chronic signs of senescence in response to TBI. As DNA damage is central to ageing and senescence [33], we focused on detecting whether DNA double strand breaks were increased using γH2AX immunofluorescence. For the experiments, we selected 24 h as the early time point to facilitate comparison with available literature data for TBI-induced glial senescence and four weeks as a later stage.

Microglia are the tissue-resident macrophages of the brain, which are responsible for inflammatory responses of the brain, phagocytosing and clearing cellular debris to maintain CNS homeostasis. However, microglia also have physiological interactions with both neurons and the vasculature in the brain [34]. In response to TBI, microglia exhibit a prolonged activation and inflammatory response, which contributes to the long-term behavioural and cognitive effects of the injury [35]. Microglia senescence, which has been described in ageing and ageing-related neurodegenerative diseases, leads to their dysfunction, such as loss of phagocytic function, and can accelerate brain ageing [20]. As microglial activation and senescence both contribute to the degradation of neuron health and cognitive function, it is important to study their roles in TBI [36].

Our results revealed a significantly higher number of γH2AX-positive microglia in the neocortex in TBI (severe and mild groups) compared to the sham control 24 h after the injury. In the same samples, the hippocampus did not show statistically significant changes in response to TBI, although the number of γH2AX-positive microglia showed an increasing trend similar to the neocortex. In contrast with the 24 h time point, the senescent microglia disappeared at 4 weeks after the injury. The disappearance of γH2AX-positive microglia over time could be explained by multiple mechanisms, such as clearance of senescent cells; another possibility would be that the cells resolved the observed DNA damage and did not undergo senescence. A definitive answer requires further study.

Aligning with our early 24 h time point, Tominaga and colleagues reported, using immunofluorescence co-labelling, that besides neurons, microglia express increased levels of p21 after TBI in a mouse model of controlled cortical impact (CCI) [37]. According to their immunohistochemistry results, the total number of p21-positive cells increased at 1 day after injury. However, they found that the p21-positive cell number peaked at 7 days but was still high at 14 days, suggesting that senescent microglia are present for multiple days after TBI. They reported similar changes in *p21* mRNA levels over time in the injured hemisphere and also an increased expression of SA-β-gal in cells adjacent to the lesion after 4, 7 and 14 days post injury. Similar to the above mRNA data, Ritzel and colleagues reported increased *p21* mRNA and protein levels in a CCI model 3 days after injury [38]. Interestingly, using flow cytometry, they found an increased level of p16 but not p21 in microglia 3 days after injury. Taken together, these results demonstrated that experimental TBI caused a rapid increase in senescence markers in microglia regardless of the type of trauma. Discrepancies in the expression of specific senescence markers between experiments underline the finding that all senescence markers in use play important roles in other cellular processes, and understanding their interactions requires further research.

Astrocytes are among the most abundant glial cells that interface with every functional element of the brain. The senescence of astrocytes, or astrosenescence, causes the loss of proliferative ability and loss of neuronal trophic support, resulting in the inability to protect neurons [22,39]. In response to tissue deformation and tissue damage caused by TBI, astrocytes undergo reactive astrogliosis while some astrocytes undergo senescence. Even though reactive astrocytes are proliferative and senescent astrocytes are not, the two phenotypes have common morphological, protein expression and secretory features, e.g., the secretory phenotype of senescent astrocytes overlaps with that of activated astrocytes in astrogliosis [23,40]. Thus, it is important to study their respective roles in TBI [41].

In our experiments, TBI increased the number of γH2AX-positive astrocytes in the neocortex similarly to microglia, although this was significant only in the sTBI group. In the hippocampus there were negligible changes, indicating that the neocortex was more vulnerable to senescence in comparison to the hippocampus in the acute phase of the injury. Senescence of astrocytes shortly after TBI was also reported by Tominaga and colleagues using the CCI model. In their experiments, using immunofluorescence microscopy, it was detected that astrocytes expressed p16 at 7 days after the injury while neurons or microglia did not. Similar to their p21 expression results, the total number of p16-positive cells peaked at 7 days after injury and *p16* mRNA levels were also increased 4–14 days after injury [37]. Interestingly, we found that 4 weeks after the injury, all γH2AX-positive astrocytes and microglia were absent from the tissue. Senescent cells are removed by the immune system [42,43]. The macrophages that clear debris from the brain are microglia and brain-associated macrophages [44]. Recently, the meningeal lymphatic drainage pathway was also implicated in the clearance of senescent astrocytes from brain parenchyma [45]. Wang and colleagues found p16- and p21-positive astrocytes and p16-positive microglia at both 5 weeks and 4 months after CCI in mice. This may contradict the fact that we did not detect γH2AX-positive glial cells at 4 weeks post injury, though the different injury model and model animal used could account for the difference. They also reported that senolytic treatment using dasatinib combined with quercetin started 1 month after the injury and continued for 13 weeks, and significantly reduced the number of p21- or p16-positive astrocytes [46].

Brain endothelial cells create an interface between the brain tissue and the circulation. These highly specialised endothelial cells differ from their peripheral counterparts. The continuous line of tight junctions connecting brain endothelial cells, their high number of mitochondria and low number of caveolae contribute to the formation of the paracellular and transcellular barriers of the BBB. Thus, endothelial dysfunction has profound effects on both the nutrient supply and waste removal of neurons. As the BBB is the forefront defence line of the CNS thatrestricts the free movement of solutes and cellular elements between the systemic circulation and neural tissue, it is involved in the pathogenesis of a large number of CNS disorders. In most mammals, the proliferative capacity of brain endothelial cells is limited, making these cells prone to senescence [47]. Vascular cell senescence impairs BBB integrity and alters tight junction proteins [18].

In our experiments, approximately 10% of hippocampal endothelial cells were γH2AX-positive at 24 h after TBI, though due to individual variability, these results were not statistically significant. At the same time, neocortical endothelial cells did not become senescent at the earlier (24 h) time point after injury. At the later (4-week) time point, the number of γH2AX-positive endothelial cells remained unchanged in the hippocampus, but increased in the neocortex, where such γH2AX-positivity increase reached statistical significance in the sTBI group. Considering the importance of endothelial cells in brain homeostasis, it is surprising that we found no prior literature-based evidence of endothelial senescence in TBI. A recent study by Kiss and colleagues [48], using single-cell RNA sequencing, reported that 10% of cerebromicrovascular endothelial cells underwent senescence in 28-month-old mice, which was comparable to what we observed in response to TBI. Eliminating senescent cells after whole brain irradiation restores the decreased microvascular density [49]. Removed senescent endothelial cells are replaced by new ones to maintain vascular function in the brain; one source for this process originates from endothelial progenitor cells (EPCs). In response to TBI, EPC numbers in the circulation were found to be decreased at 24 h after injury, but reached a peak at 7 days after injury [50]. Senolysis in mice can increase the number of EPCs that adhere to the brain vasculature and also the integration of EPCs into microvessels [51]. Our above finding that endothelial senescence caused by TBI was comparable to what Kiss et al. found in old mice may have important implications in the outcome of TBI. TBI-induced senescence may play a crucial role in accelerating vascular ageing, possibly as the cause of bilateral microvascular rarefaction and structural changes in brain blood vessel walls observed in TBI that are normally associated with ageing [52,53].

Pericytes are important components of the NVU. They play a key role in regulating cerebral blood flow and are associated with BBB formation and maintenance. Based on in vitro data, it was expected that pericyte senescence could contribute well to TBI-induced degradation of the BBB [54].

In contrast to what we observed in endothelial cells and glial cells, we found that pericytes were resistant to TBI-induced senescence in both the neocortex and hippocampus. This latter observation is also in line with the lack of literature-based evidence of brain pericyte senescence in response to TBI, although other pericyte responses have been described after brain injury. Zehendner and colleagues [55] observed a sharp decline in the immunofluorescence signal of PDGFRβ in the pericontusional zone of mouse brain sections at 6 h after CCI in mice. The PDGFRβ immunofluorescence signal increased over the following 3 days and significantly exceeded the control at 5 days post injury. The authors interpreted these changes as changes in pericyte number, which was supported by masses of proliferating PDGFRβ-positive cells in the pericontusional zone. Similar results were observed by Whitehead and colleagues [56] in a mild–moderate TBI mouse model. Pericyte coverage, defined as immunofluorescence co-localisation of CD13 with lectin-labelled brain microvasculature, decreased after mTBI at both 7 and 14 days post injury, and was observed to recover after 28 days. In our model, we saw no evidence of decreased pericyte numbers or PDGFRβ-positive cell masses after.

With the caveat that senescence markers are in fact markers of multiple cellular processes, our results expand the available experimental data on senescence-associated markers in less studied cell types of the NVU, and especially for microvascular cells. The literature data and our results suggest that previously reported positive effects of senolytic treatment after TBI could be exerted through the decreased ratio of senescence in multiple cellular components of the NVU [57]. Insight into the dynamics and cell-type specificity of TBI-induced senescence holds promise for advancing our knowledge of TBI pathology and developing innovative treatments. An early senolytic intervention might work through the senolysis of glial cells, and a later treatment could target endothelial cells, resulting in further improvement.

## 5. Conclusions

In conclusion, based on the ratio of γH2AX-positive cells and the previous literature data found using other markers, the cellular components of the NVU showed differential senescence response, though this did not manifest in altered brain function evidenced by mNSS scoring. Astrocytes and especially microglia had an initial increase in the number of γH2AX-positive cells. The decrease or loss of glial function via senescence is a likely candidate for causing secondary damage. It remains to be seen whether and how the early senescence and the expected functional loss and SASP of glial cells may contribute to the increased ratio of γH2AX-positive endothelial cells that we observed at 4 weeks after TBI.

## Figures and Tables

**Table 1:** Primary antibodies.

Primary Antibody	Catalogue No.	Application
anti-gamma H2A.X phospho S139, mouse monoclonal	ab26350 (Abcam, Cambridge, United Kingdom)	1:100
anti-PECAM-1/CD31, goat polyclonal	AF3628 ( Bio-Techne R&D, Budapest, Hungary)	1:200
anti-PECAM-1/CD31, rabbit polyclonal	NB 100-2284 (Novus Biologicals, Centennial, CO, USA)	1:100
anti-Iba1, goat polyclonal	ab5076 (Abcam, Cambridge, United Kingdom)	1:200
anti-GFAP, rabbit polyclonal	ab7260 (Abcam, Cambridge, United Kingdom)	1:200
anti-PDGF receptor beta, rabbit monoclonal	3169S (Cell Signalling Technology, Danvers, Massachusetts, USA)	1:200

**Table 2:** Secondary antibodies.

Secondary Antibody	Catalogue No.	Application
donkey anti-goat A488 (green) (for CD31 24 h sections)	A32814 (Invitrogen)	1:500
donkey anti-mouse A647 (far red) for γH2AX	A32787 (Invitrogen)	1:500
donkey anti-rabbit A488 (green)	A32790 (Invitrogen)	1:500

## Data Availability

Data are available on request.

## Supplementary Materials

The following supporting information can be downloaded at: https://www.mdpi.com/article/10.3390/biom16030359/s1, Table S1: Details of mNSS scoring.

## Abbreviations

The following abbreviations are used in this manuscript:

BBB	blood–brain barrier
NVU	neurovascular unit
TBI	traumatic brain injury
sTBI	severe traumatic brain injury
mTBI	mild traumatic brain injury
rmTBI	repetitive mild traumatic brain injury
mNSS	modified neurological severity scoring
