# Supplementary material for: Traumatic Brain Injury Induces Senescence in Brain Microvasculature
Source: Biomolecules. 2026 Feb 28;16(3):359. doi: 10.3390/biom16030359 (PMC13024561; doi:10.3390/biom16030359)
Supplement: Supplementary file 1 [file biomolecules-16-00359-s001.zip › biomolecules-4115342-supplementary.pdf]

Supplementary table S1. Details of mNSS scoring

**Forelimb flexion reflex**	forelimb extension – 0 p<br>forelimb bending – 1 p
**Hindlimb flexion reflex**	hindlimb extension – 0 p<br>hindlimb bending – 1 p
**Head deviation test**	>10° head deviation – 1 p<br>deviation is absent – 0 p
**Gait test**	normal gait – 0 p<br>unstable gait – 1 p<br>circling – 2 p<br>falling – 3 p
**Placement tests (visual&tactile)**	touching the surface – 0 p<br>touching response is absent – 1 p
**Proprioceptive test**	paw placement response is present – 0 p<br>not present – 1 p
**Beam balance test**	0 - stable body position<br>1 - gripping to the edge of the bar<br>2 - one limb hanging off the bar<br>3 - two limbs hanging off the bar<br>4 - falling off the bar after more than 40 s<br>5 - falling off the bar after more than 20 s but less than 40 s<br>6 - fall from the bar after less than 20 s
**Pinna reflex**	reflex is present – 0 p<br>not present – 1 p
**Cornea reflex**	blinking is present – 0 p<br>not present – 1 p
**Startle reflex**	startle reflex to a loud noise – 0 p<br>reflex not present – 1 p
**Abnormal movements/muscle tone**	seizures, myoclonus or dystonia are present - 1 p<br>not present - 0 p
